# Canine Mesenchymal Cell Lyosecretome Production and Safety Evaluation after Allogenic Intraarticular Injection in Osteoarthritic Dogs

**DOI:** 10.3390/ani11113271

**Published:** 2021-11-15

**Authors:** Michela Mocchi, Elia Bari, Silvia Dotti, Riccardo Villa, Priscilla Berni, Virna Conti, Maurizio Del Bue, Gian Paolo Squassino, Lorena Segale, Roberto Ramoni, Maria Luisa Torre, Sara Perteghella, Stefano Grolli

**Affiliations:** 1 Department of Drug Sciences, University of Pavia, Viale Taramelli 12, 27100 Pavia, Italy; michela.mocchi@unipv.it (M.M.); elia.bari@unipv.it (E.B.); sara.perteghella@unipv.it (S.P.); 2 Istituto Zooprofilattico Sperimentale della Lombardia e dell’Emilia Romagna, 25124 Brescia, Italy; silvia.dotti@izsler.it (S.D.); riccardo.villa@izsler.it (R.V.); 3 Department of Veterinary Medical Science, University of Parma, 43121 Parma, Italy; priscilla.berni@unipr.it (P.B.); virna.conti@unipr.it (V.C.); roberto.ramoni@unipr.it (R.R.); stefano.grolli@unipr.it (S.G.); 4 Freelance Veterinary Medical Doctor, 43121 Parma, Italy; mauriziodelbue@gmail.com; 5 Veterinary Practitioner, Studio Tecnico Veterinario, Via Alfredo Catalani 12/14, 14100 Asti, Italy; squassino@yahoo.it; 6 Department of Pharmaceutical Sciences, University of Piemonte Orientale, Largo Donegani 2/3, 28100 Novara, Italy; lorena.segale@uniupo.it; 7 PharmaExceed S.r.l., Piazza Castello 19, 27100 Pavia, Italy

**Keywords:** mesenchymal stem/stromal cells, MSC-secretome, canine regenerative medicine, osteoarthritis

## Abstract

**Simple Summary:**

Recently, mesenchymal stromal cells have been proposed as a valid approach for treating osteoarthritis diseases, mainly due to the secretion of soluble factors and extracellular vesicles known as secretome. In this paper, an injectable freeze-dried pharmaceutical powder containing canine mesenchymal stromal cells-secretome (Lyosecretome) has been formulated, and in vitro potency experiments were conducted. Furthermore, the Lyosecretome was applied in dogs affected by osteoarthritis to assess its safety. Results indicated that intra-articular injection of allogeneic Lyosecretome is safe and does not induce an adverse response, thus paving the way for future studies regarding the efficacy.

**Abstract:**

In recent years, mesenchymal stromal cells (MSCs) have shown promise as a therapy in treating musculoskeletal diseases, and it is currently believed that their therapeutic effect is mainly related to the release of proteins and extracellular vesicles (EVs), known as secretome. In this work, three batches of canine MSC-secretome were prepared by standardized processes according to the current standard ISO9001 and formulated as a freeze-dried powder named Lyosecretome. The final products were characterized in protein and lipid content, EV size distribution and tested to ensure the microbiological safety required for intraarticular injection. Lyosecretome induced the proliferation of adipose tissue-derived canine MSCs, tenocytes, and chondrocytes in a dose-dependent manner and showed anti-elastase activity, reaching 85% of inhibitory activity at a 20 mg/mL concentration. Finally, to evaluate the safety of the preparation, three patients affected by bilateral knee or elbow osteoarthritis were treated with two intra-articular injections (t = 0 and t = 40 days) of the allogeneic Lyosecretome (20 mg corresponding 2 × 10^6^ cell equivalents) resuspended in hyaluronic acid in one joint and placebo (mannitol resuspended in hyaluronic acid) in the other joint. To establish the safety of the treatment, the follow-up included a questionnaire addressed to the owner and orthopaedic examinations to assess lameness grade, pain score, functional disability score and range of motion up to day 80 post-treatment. Overall, the collected data suggest that intra-articular injection of allogeneic Lyosecretome is safe and does not induce a clinically significant local or systemic adverse response.

## 1. Introduction

In the last few years, mesenchymal stem cells (MSCs) have shown promise as a therapy in the treatment of musculoskeletal diseases (MSDs), including different pathological conditions such as disorders of muscle, joint, bone, nerves, and tendons [1]. The disease burden of MSDs in veterinary medicine is considerable and represents a significant threat to animal welfare worldwide, particularly in canine and equine species [2,3]. MSDs are related to various factors, such as intensive and repetitive works, sport-related trauma, ageing, and genetic background [1]. However, the MSCs ability to undergo multi-lineage differentiation has long been considered a critical feature to justify their clinical application. Currently, it is proved that the therapeutic effect of MSCs is mainly related to the secretion of a wide variety of trophic factors, encompassed under the name of secretome [4,5], playing an important paracrine activity.

MSCs secretome is composed of a combination of soluble factors (mainly cytokines and growth factors) and insoluble extracellular vesicles, including exosomes and microvesicles. The numerous different features associated with the secretome suggest that it could be used as an MSCs substitute [5] to mediate their biological activity, including anti-inflammatory and tissue regenerative properties [6]. The use of MSC-secretome as an alternative to their parental cells brings several advantages in regenerative medicine: the use of a cell-free product would reduce the unwanted immune reaction, and the tumorigenic risk related to the MSCs proliferation and differentiation capability; it is also easier to handle, store and ensure a sterile product by filtration [7,8,9]. We recently proposed a GMP-compliant human secretome production process that allowed us to obtain a stable and effective product named Lyosecretome (freeze-dried secretome) from human adipose tissue-derived MSCs [10,11,12,13,14]; this process included an ultrafiltration step for the concentration and the purification of MSCs-derived secretome, and the following lyophilization, obtaining a powder dosage form ensuring an improved long-term stability of the secretome.

In the current work, we applied the same procedure to obtain canine Lyosecretome from adipose tissue-derived MSCs, as a candidate for in vivo testing on naturally occurring diseases, thanks to the promising regenerative potential of MSCs secretome. Canine Lyosecretome was first characterized through quality controls regarding physicochemical properties, vesicles morphology and size distribution, protein and lipid content; furthermore, the requirements for the preparation of injectable pharmaceutical dosage forms were evaluated. Next, in vitro potency tests were performed to assess proliferative effects on different cell lines. Finally, the proof of principle of the safety of the canine allogenic Lyosecretome was performed by intraarticular administration to osteoarthritic dogs. As far as we know, this study is the first canine study based on MSCs secretome in naturally occurring musculoskeletal diseases. The application of Lyosecretome to animals affected by spontaneous osteoarthritis, avoiding the use of experimentally induced disease models, could provide interesting information about the safety and feasibility of an allogenic cell-free regenerative medicine approach in companion animals that share with the owner’s environment and lifestyle [15], thus providing valuable clues for osteoarthritis therapy in the human counterpart.

## 2. Materials and Methods

### 2.1. Materials

The Lyosecretome production was made at an accredited facility to produce and control veterinary products for clinical use (Istituto Zooprofilattico Sperimentale Lombardia and Emilia-Romagna, IZLER, Brescia, Italy). The production protocol has been approved by the Italian Ministry of Health (Prot. n. 0000778 del 15/01/2020 7.1.2.0.0.0/17/2019-AGD 809). Adipose-derived canine MSCs were obtained by IZLER Biobank. All reagents for cell culture, e.g., DMEM, fetal bovine serum (FBS) and antibiotics, were purchased from Euroclone, Milan, Italy. Acetone, bovine serum albumin (BSA), mannitol and phosphatidylcholine (PC) were bought from Sigma Aldrich, Milan, Italy. Epigallocatechin gallate (EGCG), N-succinyl-Ala-Ala-Ala-p-nitroanilide and pancreatic porcine elastase were purchased from Merck Life Science, Milan, Italy. Otherwise specified, all the reagents used were of analytical grade.

### 2.2. Preparation and Characterization of Injectable Lyosecretome Formulations

Freeze-dried MSC-secretome (Lyosecretome) was prepared and characterized following and adapting previous procedures in compliance with the current standard ISO 9001 [10,11,12,13,14]. Adipose-derived MSCs (AD-MSCs) were seeded into flasks at 10,000 cells/cm^2^ and expanded until P3 with DMEM/F12 medium plus 10% *v*/*v* FBS, plus 1% *v*/*v* penicillin/streptomycin and 1% *v*/*v* amphotericin B at 37 °C and 5% CO_2_. Secretome was obtained by culturing MSCs in DMEM/F12 without FBS (FBS starvation) for 48 h. MSCs employed accomplished the requirements for clinical use in terms of cell viability, cellular identity, genomic stability (as stated by the International Society for Cellular Therapy [16]), sterility and apyrogenicity (according to Eu. Ph. 9.0, 2.6.7). Supernatants collected from the cell culture were centrifuged at 3500× *g* for 10 min to remove cell debris and apoptotic bodies. Supernatants were recovered and then ultrafiltered by tangential flow filtration (KrosFlo^®^ Research 2 i system, Spectrum Laboratories, Milan, Italy) using a filtration module with a superficial area of 235 cm^2^ and a molecular Weight Cut Off (MWCO) of 5 kDa (Spectrum Laboratories, Milan, Italy). The samples were first concentrated at 0.5 × 10^6^ cell equivalents per mL (calculated by dividing the total cell number and the concentrated mL of supernatant). Then, they were diafiltered using sterile ultrapure water as a buffer. The processed sample was then added of mannitol at a final concentration of 0.5% *w*/*v*, frozen at −80 °C and freeze-dried (Christ Epsilon 2–16 D LSCplus) at −50 °C and 8 × 10^−1^ mbar for 72 h. The obtained Lyosecretome was stored at −20 °C until use (6 months); each mg of Lyosecretome corresponds to 0.1 × 10^6^ cell equivalents (=total cell number/obtained milligrams of Lyosecretome).

#### 2.2.1. Lyosecretome Characterization

##### Proteins and Lipids Amount

Total proteins were dosed by the BCA Protein Assay Kit (Thermo Fisher Scientific, Milan, Italy) according to the manufacturer’s instructions. Briefly, the working reagent solution in a 1:1 ratio was added to each sample and incubated at 37 °C for 2 h. The optical density was measured at 562 nm with a microplate reader (Synergy HT, BioTek, United Kingdom). Standard protein solutions (BSA) were used to build a calibration curve (R^2^ = 0.99). Total lipids were dosed by the Nile Red method, which was previously validated [10]. The Nile Red powder was solubilized in acetone at a concentration of 3.14 M and then diluted 100× in PBS. Nile Red solution was added to samples (1:9 ratio) and incubated for 5 min at room temperature. The fluorescence was measured by Synergy HT at 530/25 excitation and 645/40 emission. Standard lipid solutions (PC) were used to build a calibration curve (R^2^ = 0.99). All analyses were done in triplicate.

##### EV Particle Size and Concentration

Nanoparticle Tracking Analysis (NTA, NanoSight NS 300 equipment, Malvern Instruments, Malvern, UK) was used to measure EVs’ particle size and concentration into Lyosecretome. The freeze-dried powder was analyzed at room temperature at the concentration of 1 mg/mL in deionized water. By adjusting the instrument setting, raw data were processed by the NTA software v3.2 (Malvern Instruments, Malvern, UK). All analyses were done in triplicate.

##### Physical-Chemical Characterization

Fourier-Transform Infrared Spectroscopy (FT-IR) spectra of the Lyosecretome were evaluated between 650 and 4000 cm^−1^ spectral regions with a resolution of at least 4 cm^−1^. A Spectrum One Perkin-Elmer spectrophotometer (Perkin Elmer, Wellesley, MA, USA) arranged with a MIRacle™ ATR device (Pike Technologies, Madison, WI, USA) was exploited. All measurements were done in triplicate. Differential Scanning Calorimetry (DSC) analysis was performed with a Mettler STAR^e^ system (Mettler Toledo, Columbus, OH, USA) equipped with a DSC821 e Module and an Intracooler device (Jukabo FT 900) (JULABO GmbH, Seelbach, Germany) for sub-ambient temperature analysis. The instrument, after calibration with Indium as a standard, was used on samples; the curves were recorded on about 3 mg in 40 µL sealed aluminium pans with pierced lid. The following parameters were considered: temperature range −30–250 °C; heating rate 10 K min^−1^; nitrogen air atmosphere flux 50 mL min^−1^). Each experiment was performed in triplicate. Thermo Gravimetric Analysis (TGA) was performed with a Mettler STARe system (Mettler Toledo, Columbus, OH, USA) arranged with a TGA/DSC1 (Mettler Toledo, Columbus, OH, USA). The instrument was previously calibrated with Indium as a standard reference; then, the curves were recorded on about 4 mg of samples in 70 µL alumina pans. The following parameters were considered: temperature range 30–300 °C; heating rate 10 K min^−1^; nitrogen air atmosphere flux 50 mL min^−1^. Each experiment was performed in triplicate.

##### Sterility and Apyrogenicity

Sterility, endotoxins, mycoplasma and other microbiological contaminations were evaluated on a representative sample using the same tests applied to ensure the sterile conditions of the starting cells. In detail, for microbial control, the samples were examined through a microbiology test according to the provisions of the European Pharmacopoeia (EuPh 2.6.2). Apyrogenicity was ensured by quantitatively detecting bacterial endotoxins using the Limulus Amebocyte Lysate (LAL) test (EuPh 2.6.14) and measured as Endotoxin Unit (EU). Possible mycoplasma contamination was detected by carrying out specific tests (NAT test) following the provisions of European Pharmacopoeia (EuPh 2.6.7).

### 2.3. In Vitro Potency and Efficacy Tests

#### 2.3.1. Specimen Collection

Adipose tissue, tendon, and cartilage tissue samples were collected from animals at the local Veterinary Teaching Hospital. All animal owners were aware of the study and signed an informed consensus, and the protocols were approved by the Italian Ministry of Health (Prot. n. 0000778 del 15/01/2020 7.1.2.0.0.0/17/2019-AGD 809). All the animals were subjected to a complete clinical examination, blood count (CBC), and serum biochemistry analysis. Tendons and cartilage were collected two to six hours after the death from the knee joint of animals who died of natural causes and were subjected to necroscopy. Patients affected by infectious diseases were excluded.

#### 2.3.2. MSCs Isolation from Adipose Tissue, Tendon and Cartilage

Once collected, each type of tissue was washed in 70% ethanol and stored in phosphate-buffered saline (PBS) containing penicillin (50 U/mL), streptomycin (20 μg/mL), and amphotericin B (2.5 μg/mL) for a maximum of 2h before being processed. Then, 1–1.5 g of tissue were fragmented in 0.3–0.5 cm^3^ pieces in a Petri dish with scalpels and sterile pliers. The fragments were transferred to a 15 mL conical centrifuge tube containing a solution of 0.1% *w*/*v* collagenase type I, prepared in DMEM, supplemented with 100 U/mL penicillin, 100 μg/mL streptomycin, 2.5 μg/mL amphotericin B, at a ratio of 5 mL of medium per gram of minced tissue. The samples were enzymatically digested in a water bath at 37 °C, in moderate agitation, for 1 h for adipose tissue and 2 h for tendon and cartilage fragments. The solution was then filtered with a nylon filter (mesh 100 μm) and centrifuged at 190× *g* for 15 min. The pellet obtained was followed by resuspension in 2 mL of maintenance medium (mDMEM), consisting of DMEM supplemented with 10% *v*/*v* FBS, penicillin 100 U/mL, streptomycin 100 μg/mL, amphotericin B 2.5 μg/mL, and then seeded in 25 cm^2^ cultures flasks. The cells were maintained in an incubator at 37 °C in a 5 % CO_2_ atmosphere, renewing the medium every 72 h. Once 80% confluence was reached, the cells were detached with 0.05% Trypsin-EDTA in PBS and expanded to P3–P4 when used for the experiments described below.

#### 2.3.3. Cell Metabolic Activity Evaluation

The cytocompatibility and proliferation ability of Lyosecretome were evaluated on three different cell lines: canine MSCs, canine tenocytes and canine chondrocytes. Cells were seeded in 96-well plates at 10,000 cells/well for 24 h and treated with increasing doses of Lyosecretome (50,000, 100,000 and 200,000 cell equivalents/well) in a serum-free medium. Cells not treated with Lyosecretome or treated with 10% *v*/*v* FBS were considered negative and positive control. After 48 h, an MTT test was performed. The absorbance was measured by a microplate reader (Victor Nivo, Perkin Elmer, Waltham, MA, USA) at 570 nm and 670 nm (reference wavelength), and the metabolic activity percentage was calculated as:

Cell metabolic activity (%) = 100 × (Abs_sample_/Abs_positive control_). (1)

#### 2.3.4. Anti-Elastase Activity

The suppressive effect of Lyosecretome on pancreatic porcine elastase was assessed in vitro. Briefly, the enzyme was solubilized in phosphate buffer pH 6.8 at 0.5 IU mL^−1^. Simultaneously, the substrate consisting of N-succinyl-Ala-Ala-Ala-p-nitroanilide was dissolved at a final concentration of 0.41 mmol L^−1^ in TRIS buffer. Increasing concentrations of Lyosecretome (2, 5, 10, 20 mg mL^−1^) were incubated at room temperature with the enzyme for 20 min; then, the substrate was added, and the kinetic reaction was monitored by spectrophotometric analysis (Synergy HT) at the absorbance of 410 nm for 35 min (one measurement/minute). The reaction mixture in the absence of Lyosecretome was used as a negative control, while EGCG at 7.2 mg/mL was used as a positive control. Analyses were performed in triplicate, and the percentage anti-elastase activity (%) was calculated as:

Anti-elastase activity (%) = [(A_CTR_ − A_samp_) / A_CTR_] × 100 (2)

where A_CTR_ is the optical density of the negative control, and A_samp_ is the optical density of the Lyosecretome sample.

### 2.4. In Vivo Safety Evaluation

In vivo safety of Lyosecretome was evaluated on dogs affected by naturally occurring osteoarthritis (OA).

#### 2.4.1. Ethical Concerns

The clinical trial has been approved by the Italian Ministry of Health (Prot. n. 0000778 del 15/01/2020 7.1.2.0.0.0/17/2019-AGD 809). The clinical experimentation has also been approved by the ethical committee of Istituto Sperimentale della Lombardia ed Emilia Romagna (IZSLER, n. 1/2019, 21 March 2019).2.4.2. Animal Recruiting and Inclusion/Exclusion Criteria.

The current study enrolled 5 client-owned dogs presenting bilateral elbow or knee osteoarthritis (Table 1). The animals were treated with two intra-articular injections of Lyosecretome at 40-days intervals. The owners of all the animals involved in the study signed a written donor consent after being notified of the relevant project information. For the enrollment, the patients underwent a complete clinical examination, complete blood count and serum biochemistry analysis to exclude other pathologies, followed by an orthopaedic examination to confirm and grade the OA. Details on the different clinical cases are reported in the Supplementary materials (Section S1, Figures S1–S3).

This study evaluated the following orthopaedic parameters: lameness, pain, functional disability, range of motion, and OA grade (Table 2, Table 3 and Table 4). A disease score was obtained by adding scores for each of these parameters, ranging from grade 1 to 5 (Table 5). Only Grade 5 dogs were enrolled in this study.

#### 2.4.2. Pre-Treatment and Post-Treatment Evaluations

A full orthopaedic examination was conducted with X-rays under sedation to assess the baseline severity of OA in each animal. Radiographic signs of OA were evaluated using the Kellgren–Lawrence scale for the knee joint [17] and the International Elbow Working Group (IEWG) guidelines for the elbow joint [18]. The OA was categorized as shown in Table 3 and Table 4. The orthopaedic examination of the animals included the assessment of lameness grade, pain score, functional disability score and range of motion (ROM) on day 0 (t0) and 40 days after each intra-articular treatment (t40 and t80). In detail, to assess the lameness score, gait was observed at stand, walk and trot and recorded by video, and lameness was categorized from grade 1 to 5 (Table 2) [19]. Each joint was then manipulated for pain and functional disability, and the ROM was assessed with an orthopaedic goniometer, giving a score from 1 to 5 to each parameter (Table 2) [17,19].

The follow-up included an owner questionnaire (Table 6) containing the validated Helsinki chronic pain index (HCPI) for up to 80 days (t0, t2, t4…t20, t40, and t80). The questionnaire inquired about the dog’s general condition (body temperature, appetite) and local reactions in the treated joints (heat, pain, and joint swelling). Owners were also asked to report any abnormal response of the patient or NSAID treatment needed. Chronic pain signs were assessed with the HCPI that contained questions regarding the dog’s mood, lameness, and willingness to move, play, and jump. The HCPI included 11 questions, whose answers were given by a score of 0 to 4, where 0 and 1 indicate normal behaviour and movement, while 2, 3, and 4 indicate pain with increasing severity [20,21,22]. The owner questionnaire based on HCPI and additional information was used to assess animal welfare before the treatment and to collect local or systemic adverse reactions, providing information about the safety of the treatment.

#### 2.4.3. Intra-Articular Injection of Lyosecretome

After examination, dogs were sedated, and the skin over the joint was prepared for a sterile invasive procedure. Two product stocks were prepared by suspending Lyosecretome (20 mg corresponding to 2 × 10^6^ cell equivalents) or mannitol (placebo) in 1 mL hyaluronic acid (Athenavis 1%, Ibn Savio, Pomezia, Italy), containing 20 mg of sodium hyaluronate, 800–1300 KDa, in 2 mL,). For each animal, the right joint was infiltrated with the same stock and the left joint with the other stock, both times. Both the veterinarian and the owner were unaware of which treatment the joint received. In detail, a 20-gauge needle was introduced into the joint space, synovial fluid was aspirated, and the product was injected through the same needle and a syringe of 2.5 mL. At the end of the infiltration, the joints were subjected to slight flexion-extension movements to promote the spread of the product. After the procedure, the patient was sent home, prescribing rest for the first two days, resuming regular activity from the third day. The use of any anti-inflammatory medication was prohibited during the entire course of the 180 days study except in exceptional circumstances. The orthopaedic evaluation and the owner questionnaire were performed to evaluate the response to treatment and compare it with the initial clinical condition.

### 2.5. Statistical Analysis

STATGRAPHICS XVII (Statpoint Techonologies, Inc., Warrenton, VA, USA) program was used to analyzed raw data producing a general linear analysis of variance model (ANOVA). The function was then followed by an LSD test to estimate the statistical differences between means. In detail, each batch was evaluated as the variable response protein and lipid content, and the batch number was assumed as the fixed factor. The cell metabolic activity was set as the response variable and the Lyosecretome concentration as a fixed factor to evaluate cell proliferation. The anti-elastase activity data were elaborated considering the batch, concentration, and time as fixed factors, and the activity % as the response variable. Statistical significance was determined at *p* < 0.05.

## 3. Results and Discussion

Injectable Lyosecretome (freeze-dried secretome) formulations have been prepared by standardized processes under ISO9001 clinical grade. Overall, three batches were produced starting from three different cell lines, as summarized in Table 7.

After ultrafiltration to concentrate and purify the cell culture supernatant, the product was added to the cryoprotectant and freeze-dried. A filtering module with a 5 kDa molecular weight cut-off was used to retain in the ultrafiltrate both extracellular vesicles (EVs) and proteins with weight above the cut-off. The use of this membrane size was justified by preliminary investigations, demonstrating a higher immunomodulatory potency in 5 kDa ultrafiltrate (containing both EVs and soluble factors) instead of the 300 kDa ultrafiltrate (containing only EVs) [10]. Furthermore, Bari et al. [11] demonstrated that the concentration of IL-6 was higher in samples prepared by ultrafiltration rather than ultracentrifugation. Since IL-6 is a key player in the immunomodulatory features of the MSCs secretome, the large amounts of IL-6 discarded in the ultracentrifugation procedure suggest ultrafiltration is an effective procedure to prepare EV fraction. Similarly, higher anti-elastase activity was demonstrated for the whole secretome and attributed to the large amount of Alpha-1-Antitrypsin, the main inhibitor of neutrophil elastase, in the soluble fraction of the secretome [14]. Accordingly, Mitchell and colleagues found that EVs and soluble molecules act synergistically to promote tissue regeneration [23], supporting low cut-off ultrafiltration as a valuable process to prepare MSCs secretome.

The pharmaceutical quality of Lyosecretome was defined by setting up a complete characterization of total protein and lipid content, physical-chemical properties, and particle size. The amount of proteins and lipids for each batch produced is reported in Table 8. It is worth noting that no systematic error influenced the calibration for both assays as the intercept of the curve equation is not statically significant, and the plot of the residuals had an ordinary distribution of the error (data not shown). Overall, the yield of each batch is different, meaning that the cell line and, more in general, the variability within the same species can strongly influence the secretome composition in terms of protein and lipid quantity (*p* < 0.05). Maximum protein production was obtained in batch n. 1; by contrast, lipid amount is higher in batch n. 2. This aspect underlines that there is no correlation between the two assays, probably depending on cell density at the starvation time that can influence secretome production.

The physical-chemical characterization confirmed the simultaneous presence of both proteins and lipids in the Lyosecretome. In detail, FTIR spectra revealed low-intensity bands at around 1653 cm^−1^ and 1547 cm^−1^ (amide I C=O stretching vibrations and amide II N-H bending vibrations of the peptide groups, respectively), absorbance bands at about 1457 cm^−1^ and 1377 cm^−1^ (CH_2_ and CH_3_ groups) and 1260 and 880 cm^−1^ (due to the stretching vibrations typical of phospholipids, triglycerides and cholesterol esters, and to the vibrations bands of mannitol) [10,11]. By DSC and TGA analysis, it was instead confirmed that the lyophilization process occurred successfully (data not shown).

**Figure 1:**
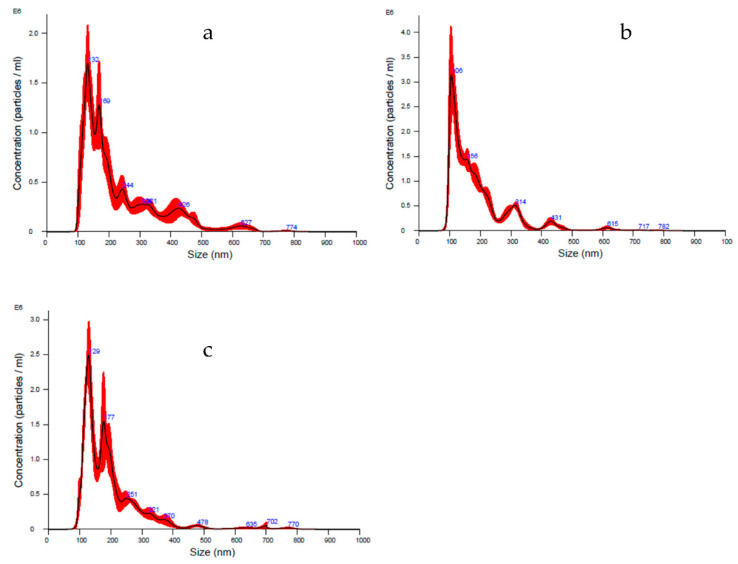
NTA averaged curves as particles concentration and size distribution of batch 1 (**a**), 2 (**b**), and 3 (**c**), respectively. The averaged curve was generated by processing six cycles of 60 s each per batch. Concentration is indicated as mean value ± standard deviation (*n* = 3). The red part shows the ± standard error of the mean values (*n* = 3).

Lyosecretome particles’ size distribution and characterization were evaluated using NTA technology. In detail, the diameter ranges between 190–230 nm, matching the choice to consider both exosomes (40–120 nm) and microvesicles (250–1000 nm). Furthermore, a heterogeneous population is shown by means analyzing curve, whereas d_10_ is set between 106–122 nm, d_50_ is set between 157–180 nm, and d_90_ is set between 316–400 nm. Thanks to this technique, the particle concentration per mL was estimated, ranging between 1.86 × 10^8^ and 2.60 × 10^8^ particle/mL (Table 9 and Figure 1).

Finally, since the Lyosecretome production was aimed at in vivo application, to guarantee the quality of the final product, microbiological tests were conducted before and after lyophilization, thus, on both secretome solution and lyophilized secretome (Lyosecretome) powder. Analyses were performed at a certified institute (IZSLER, Brescia, Italy), and the sterility conditions were ascertained in the same establishment. The results proved the absence of any mycoplasma and bacterial endotoxin level in each examined sample (data not shown).

**Figure 2:**
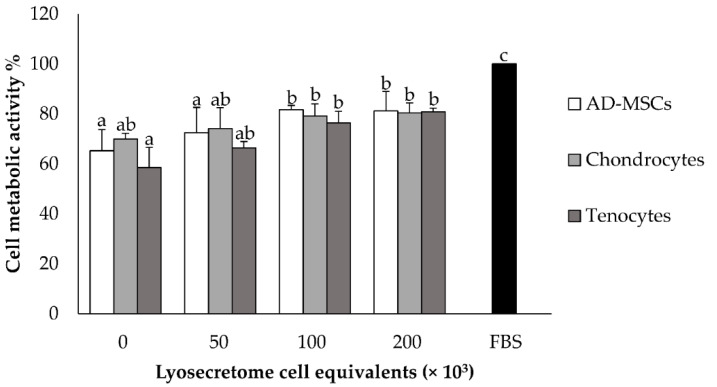
The dose-dependent trend of cell metabolic activity on AD-MSCs, chondrocytes and tenocytes tested at the Lyosecretome concentrations of 50, 100, 200 cell equivalents × 10^3^/well. 10% *v*/*v* FBS was considered positive control, while culture media without Lyosecretome and FBS were negative control (0). Multifactor ANOVA, mean values ± LSD (*n* = 3). Different letters (a, b and c) indicate a significant difference between the means (*p* < 0.05), while the same letters indicate no significant differences (*p* > 0.05).

Regarding the in vitro efficacy, Lyosecretome proliferation potency was assessed by MTT assay. Canine tenocytes, chondrocytes and adipose tissue-derived MSCs were chosen as target cells, the latter because the tissues resident MSCs can mediate tissue regeneration [24]. Lyosecretome stimulated cell metabolic activity in a dose-dependent trend for all cell types; in detail, the treatment with higher Lyosecretome concentrations reached almost 85% of cell metabolic activity compared to 10% FBS supplemented cultures. As shown in Figure 2, the dose-dependent effect was evaluated up to 200,000 cell equivalent/well. The effect of Lyosecretome treatment was statistically different for the three cell types (*p* < 0.0001).

**Figure 3:**
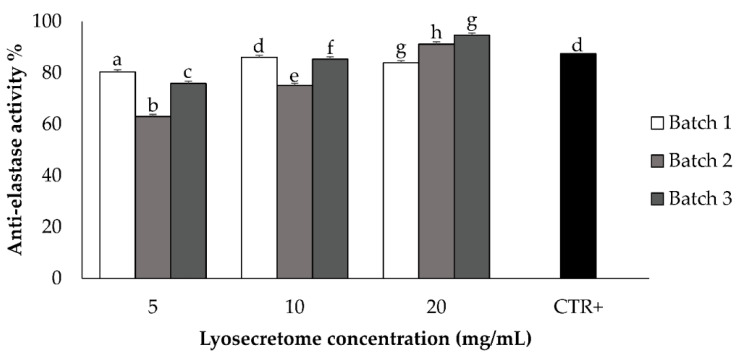
The anti-elastase activity was tested for the three batches at the concentrations of 2, 5, 10 and 20 mg/mL of Lyosecretome. A dose-dependent trend was obtained. CTR + was epigallocatechin gallate at a concentration of 7.2 mg/mL in deionized water. Multifactor ANOVA, mean values ± LSD (*n* = 3). Different letters (a–h) indicate a significant difference between the means (*p* < 0.05), while the same letters indicate no significant differences (*p* > 0.05).

The anti-elastase activity of canine Lyosecretome demonstrated a dose-dependent trend for all the tested concentrations of 2, 5, 10 and 20 mg/mL (Figure 3). The assay consists of an enzymatic reaction which was evaluated for up to 40 min. At the higher concentration of 20 mg/mL, the activity reached 85%, considering Epigallocatechin gallate as a positive control due to its inhibition of elastase activity.

Despite this study has not performed a qualitative analysis of protein, RNA, and lipid contents of canine Lyosecretome, proteomic analyses of equine [2] and human [10] Lyosecretome revealed proteins involved in controlling the inflammatory pathways activated in osteoarthritic joints and proteins related to cartilage biology. This suggests using Lyosecretome as a substitute for cells in treating musculoskeletal system diseases [25]. Furthermore, microvesicles are considered safer both from the point of view of immuno-reactivity and for the lower potential risks compared to the administration of in vitro expanded cells [26]. Still, before testing Lyosecretome efficacy, the in vivo safety must be evaluated. In this regard, this study enrolled five dogs affected by bilateral OA to test Lyosecretome for clinical use. One dog was excluded from the beginning for recent nonsteroidal anti-inflammatory drugs (NSAIDs) treatment (Animal 5, Table 1), and another dog was excluded before the second administration of the treatment for a recurrent skin infection that could negatively affect the intra-articular treatment (Animal 4, Table 1).

Lyosecretome and a placebo, consisting of mannitol (used in Lyosecretome formulation as a stabilizer), were resuspended in hyaluronic acid and injected in each animal’s right and left joint, respectively. The choice to treat both affected joints with hyaluronic acid, whose lubricating properties support its widespread use in treating OA in dogs [27], was made to ensure effective therapy in the control of clinical symptoms for patients enrolled in the study. The data collected with the questionnaire (Table 6) and reported in Table S1 have been analyzed to evaluate the onset of undesired effects following therapy (see Supplementary materials, Figures S4 and S5). No systemic adverse reactions were observed, even after the second administration. After each treatment, short-time side effects were observed for all the patients’ as reluctance to trot or gallop. However, these adverse effects resolved within two days without the need for additional treatment. Since the administration of Lyosecretome (in the right joint) and placebo (in the left joint) co-occurred, it was impossible to attribute the observed symptoms to one of the two treatments. Similar sides effects were also observed by Lee et al. [28]; the authors reported that following hyaluronic acid intraarticular injection, a dog showed a mild systemic inflammatory reaction, and two dogs showed non-weight-bearing lameness.

Only patient 3 was affected by swelling at the level of both the right and left joints (See Supplementary Figure S2) accompanied by complaining and groaning and not ease of movement for both lying down and moving after a long rest period. Thus, patient 3 likely showed an adverse reaction that was not directly attributable to the administration of Lyosecretome, as the same response was evidenced even when administering the placebo. Furthermore, the reported effects are commonly noticed immediately after intra-articular injections when animals’ lameness score is enhanced [29,30]. Finally, regarding the clinical data, the examination by the veterinary practitioners, performed at days 40 and 80 after the first treatment, were used to assess the possible onset of unexpected symptoms after therapy administration. No significant results were observed in terms of lameness and pain worsening.

In conclusion, given the limited number of enrolled patients, the present work does not provide any direct evidence of the efficacy or safety of the Lyosecretome in the treatment of canine OA. Nevertheless, it is the proof-of-concept for veterinary clinical-grade MSC-secretome production; moreover, the feasibility of two consecutive allogeneic Lyosecretome intraarticular administration in osteoarthritic dogs is proved, without evidence of adverse reactions. Therefore, this paper represents the premise for patient recruitment in a safety/efficacy clinical trial, with a proper number of cases.

## Figures and Tables

**Table 1:** Summary of cases included in this study regarding the breed, age, gender, weight and affected joint.

Animal	Breed	Age (Years)	Gender	Weight	Joint
1	Labrador	8	F	35	Knee
2	Labrador	9	F	35	Elbow
3	Golden retriever	5	M	34	Elbow
4	Labrador	3	M	32	Elbow
5	Labrador	9	M	30	Elbow

**Table 2:** Overview of the score systems used for the orthopaedic examination.

Parameter	Score	Definition
Lameness	1	Stands, walks and trots normally
	2	Stands normally, slightly painful gait when trotting
	3	Stands normally, slightly painful gait when walking
	4	Stands normally, evident painful gait when walking
	5	Stands abnormally, evident painful gait when walking
Pain	1	None
	2	Mild signs
	3	Moderate signs
	4	Severe signs
	5	The dog will not allow palpation
Functional disability	1	Normal
	2	Slightly stiff
	3	Stiff
	4	Very stiff, unwilling to walk
	5	Need assistance to walk
Range of motion	1	No limitation of movement or crepitus
	2	10 to 20% decrease in range of motion, no crepitus
	3	10 to 20% decrease in range of motion with crepitus
	4	20 to 50% decrease in range of motion
	5	More than 50% decrease in range of motion

**Table 3:** The Kellgren–Lawrence grading of osteoarthritis for the knee joint.

OA	Grading	Radiographic Features
Absent	0	No abnormalities
Doubtful	1	Minute osteophytes
Minimal	2	Definite osteophytes
Moderate	3	Diminished joint space
Severe	4	Greatly diminished joint space + sclerosis of the subchondral bone

**Table 4:** International Elbow Working Group (IEWG) guidelines for elbow osteoarthritis.

OA	Grading	Radiographic Features
Absent	0	Normal elbow joint, no evidence of incongruency, sclerosis, or arthrosis
Mild	1	Presence of osteophytes < 2 mm high, sclerosis of the base of the coronoid process—trabecular pattern still visible
Moderate	2	Presence of osteophytes of 2–5 mm high, obvious sclerosis (no trabecular pattern) of the base of the coronoid processes
Severe	3	Osteophytes of over 5 mm found anywhere in the joint

**Table 5:** Disease score was obtained by adding the scores of all the parameters evaluated in this study.

Sum of Scores	Disease Score
0–2	1
3–6	2
7–10	3
11–14	4
15–20	5

**Table 6:** Questionnaire submitted to the owners. The questionnaire is based on Helsinki chronic pain index (HCPI), with additional information collected to evaluate local and systemic adverse reactions reported by the animal owners.

Question Asked	0 Points	1 Point	2 Points	3 Points	4 Points
D2. Pain upon right joint palpation	Absent	Mild	Moderate	Severe	Does not allow palpation
D3. Pain upon left joint palpation	Absent	Mild	Moderate	Severe	Does not allow palpation
D4. Swelling upon right joint palpation	Absent	Mild	Moderate	Severe	Does not allow palpation
D5. Swelling upon left joint palpation	Absent	Mild	Moderate	Severe	Does not allow palpation
D6.1 Heat in the right joint	No warmer than contralateral limb	Mildly warmer	Moderately warmer	Markedly warmer	
D6.2 Heat in the left joint	No warmer than contralateral limb	Mildly warmer	Moderately warmer	Markedly warmer	
D7. Rate your dog’s mood	Very alert	Alert	Neither alert nor indifferent	Indifferent	Very indifferent
D8. Rate your dog’s willingness to participate in play	Very willing	Willing	Reluctantly	Very reluctantly	It does not play at all
D9. Rate your dog’s complaining and groaning	Never	Hardly ever	Sometimes	Often	Very often
D10. Rate your dog’s willingness to walk	Very willing	Willing	Reluctantly	Very reluctantly	It does not walk at all
D11. Rate your dog’s willingness to trot	Very willing	Willing	Reluctantly	Very reluctantly	It does not trot at all
D12. Rate your dog’s willingness to gallop	Very willing	Willing	Reluctantly	Very reluctantly	It does not gallop at all
D13. Rate your dog’s willingness to jump (e.g., into a car, onto a sofa)	Very willing	Willing	Reluctantly	Very reluctantly	It does not jump at all
D14. Rate your dog’s ease in lying down	With great ease	Easily	Neither easily nor difficultly	With difficulty	With great difficulty
D15. Rate your dog’s ease in rising from a lying position	With great ease	Easily	Neither easily nor difficultly	With difficulty	With great difficulty
D16. Rate your dog’s ease of movement after long rest	With great ease	Easily	Neither easily nor difficultly	With difficulty	Very often/always difficulty
D17. Rate your dog’s ease of movement after major activity or heavy exercise	With great ease	Easily	Neither easily nor difficultly	With difficulty	Very often/always difficulty
D18. Dog’s appetite	Normal	Capricious	Decreased	Markedly decreased	It does not eat at all
D19. Dog’s body temperature	Normal	Fever			
D20. Use of analgesic/anti-inflammatory drugs	No	Yes			
D21. Abnormal post-treatment reactions	None	Physical tiredness	Physical tiredness + joint pain/swelling	Increased lameness	Does not stand and walk at all

**Table 7:** Lyosecretome batch report.

Batch/Cell Line	Total Cell Number (×10^6^)	Cell Viability (%)
1	190	99
2	96	98
3	92	99

**Table 8:** Total protein and lipid content in Lyosecretome batches; mean values ± standard deviation, *n* = 3. Different letters (a, b and c) indicate a significant difference between groups of the same column (*p* < 0.0001).

Batch n.	μg Proteins/mg Lyosecretome	μg Lipids/mg Lyosecretome
1	79.5 ± 0.6 ^a^	18.9 ± 0.5 ^a^
2	28.6 ± 1.5 ^b^	5.7 ± 0.1 ^b^
3	25.9 ± 0.1 ^c^	15.5 ± 0.3 ^c^

**Table 9:** Particle size distribution and concentration of batch 1, 2 3 (mean values ± standard deviation, *n* = 3).

Batch n	Mean (nm)	Mode (nm)	d10 (nm)	d50 (nm)	d90 (nm)	Concentration Particle /mL
1	231.6 ± 8.4	143.6 ± 11.7	122.3 ± 4.1	180.6 ± 5.5	403.5 ± 20.3	1.86 × 10^8^ ± 6.37 × 10^7^
2	189.1 ± 2.7	109.7 ± 3.7	106.2 ± 2.7	157.5 ± 2.8	316.6 ± 1.8	2.60 × 10^8^ ± 1.35 × 10^7^
3	197.1 ± 6.0	148.0 ± 15.4	116.7 ± 1.3	171.6 ± 6.5	319.7 ± 16.6	2.12 × 10^8^ ± 1.06 × 10^6^

## Data Availability

The data presented in this study are contained within the article.

## Supplementary Materials

The following are accessible online at www.mdpi.com/article/10.3390/ani11113271/s1, Table S1. Data collected from the questionnaire. File S1: History and clinical findings of the three dogs treated in the study. Figure S1. Cranio-caudal radiographic projection of the knee of Animal 1. Figure S2. Medio-lateral radiographic projections of the elbow of Animal 2. (A) Maximally flexed view of the right limb. (B) Maximally flexed view of the left limb. Figure S3. Medio-lateral radiographic projections of the elbow of Animal 3. (A) Maximally flexed view of the right limb. (B) Maximally flexed view of the left limb. Figure S4. Scores for questions 11 (A), 12 (B) and 21 (C) as a function of time. The treatment (Lyosecretome or placebo) was administered on day 0 and day 40 (circled). Multifactor ANOVA, mean values ± LSD (*n* = 3). * *p* < 0.05 vs t = 0; # *p* < 0.05 vs t = 2. Figure S5. Scores for questions 4 (A), 5 (B), 9 (C), 14 (D) and 16 (E) for each participant. Multifactor ANOVA, mean values ± LSD (*n* = 3). Different letters (a-h) indicate a significant difference between the means (*p* < 0.05), while the same letters indicate no significant differences (*p* > 0.05).
