# Supplementary material for: Canine Mesenchymal Cell Lyosecretome Production and Safety Evaluation after Allogenic Intraarticular Injection in Osteoarthritic Dogs
Source: Animals (Basel). 2021 Nov 15;11(11):3271. doi: 10.3390/ani11113271 (PMC8614457; doi:10.3390/ani11113271)
Supplement: Supplementary file 1 [file animals-11-03271-s001.zip › Supplementary materials.pdf]

Canine Mesenchymal Cell Lyosecretome Production and Safety Evaluation After Allogenic Intraarticular Injection in Osteoarthritic Dogs

Michela Mocchi, Elia Bari, Silvia Dotti, Riccardo Villa, Priscilla Berni, Virna Conti, Maurizio Del Bue, Gian Paolo Squassino, Lorena Segale, Roberto Ramoni, Maria Luisa Torre, Sara Perteghella and Stefano Grolli

S.1 Animals Details: History and Clinical Findings

The current study was carried out enrolling 5 client-owned dogs presenting bilateral elbow or knee osteoarthritis. One dog was then excluded for recent nonsteroidal anti-inflammatory drugs (NSAIDs) treatment. Another dog was excluded before the second treatment administration for a recurrent skin infection that could negatively affect the intra-articular treatment. Details of the three remaining animals are shown below. The animals enrolled had a grade 5 disease score, as described in paragraph 2.4.2. and Table 5.

Animal 1

An 8-year-old female Labrador Retriever weighing 35 kg was referred for chronic lameness in the left hind limb (at least three weeks). Based on the medical history, the dog underwent surgery for a cranial cruciate ligament rupture in the right limb when she was 4 years old. At the visit, radiographic examination highlighted the presence of bilateral grade-5 knee osteoarthritis related to grade-5 hip dysplasia (Figure S1). According to the orthopaedic examination at time 0 (t0), the symptoms were identified only in the left hind limb, showing a grade-3 lameness associated with mild pain and stiffness and slight limitation of the ROM.

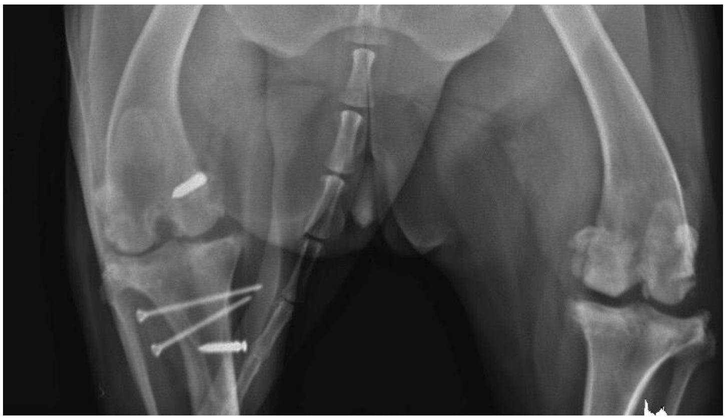

Figure S1. Cranio-caudal radiographic projection of the knee of Animal 1.

Animal 2

A 9-year-old female Labrador Retriever weighing 35 Kg was referred for chronic lameness in the right forelimb (at least three weeks). At the time of the visit, a radiographic examination highlighted the presence of bilateral grade-4 elbow osteoarthritis (Figure S2). According to the orthopaedic examination at t0, the dog presented a grade-4 lameness in the right limb associated with stiffness and mild limitation of the ROM.

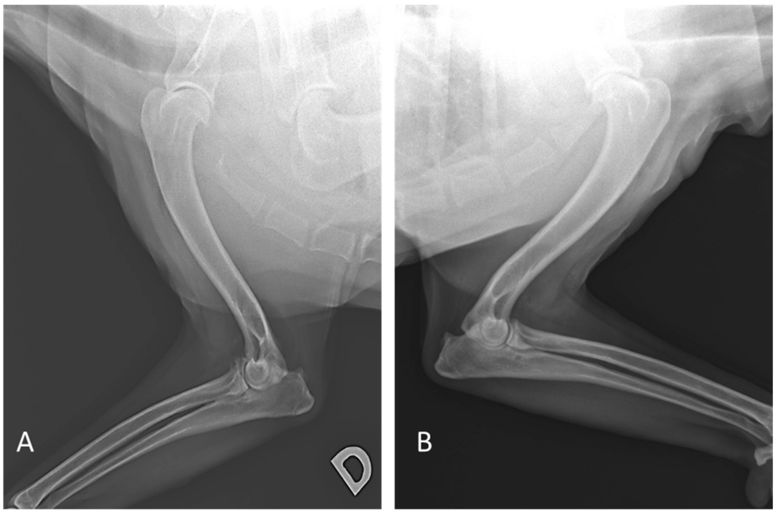

Figure S2. Medio-lateral radiographic projections of the elbow of Animal 2. (A) Maximally flexed view of the right limb. (B) Maximally flexed view of the left limb.

Animal 3:

A 5-year-old intact male Golden Retriever weighing 34 kg was referred for chronic lameness in the left forelimb (at least three weeks). Based on the medical history, the dog underwent arthroscopy for fragmentation of the coronoid process in the right limb when he was 1 year old. At the time of the visit, a radiographic examination highlighted the presence of bilateral grade-4 elbow osteoarthritis (Figure S3). According to the orthopaedic examination at t0, the dog presented a grade-3 lameness in the left limb, associated with mild pain, stiffness, and severe limitation of the ROM.

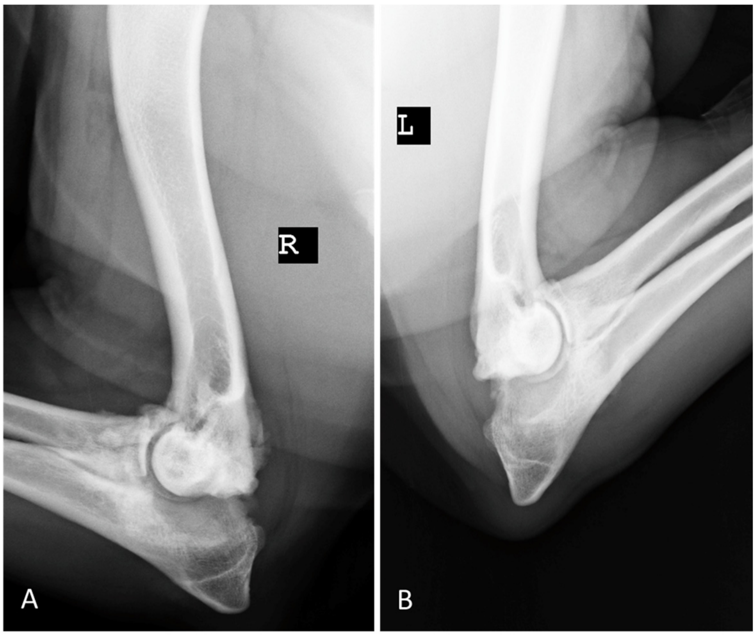

Figure S3. Medio-lateral radiographic projections of the elbow of Animal 3. (A) Maximally flexed view of the right limb. (B) Maximally flexed view of the left limb.

The data collected with the owner questionnaire (Table 6) are reported in Table S1. The questionnaire aimed to evaluate local and systemic adverse reactions reported by the owners. The data collected with the questionnaire were analyzed considering the time or the dog as a fixed factor (one-way ANOVA) and the time as co-variate (two-way ANOVA). Statistical significance was set at p<0.05. Results are reported in Figures S4 and S5.

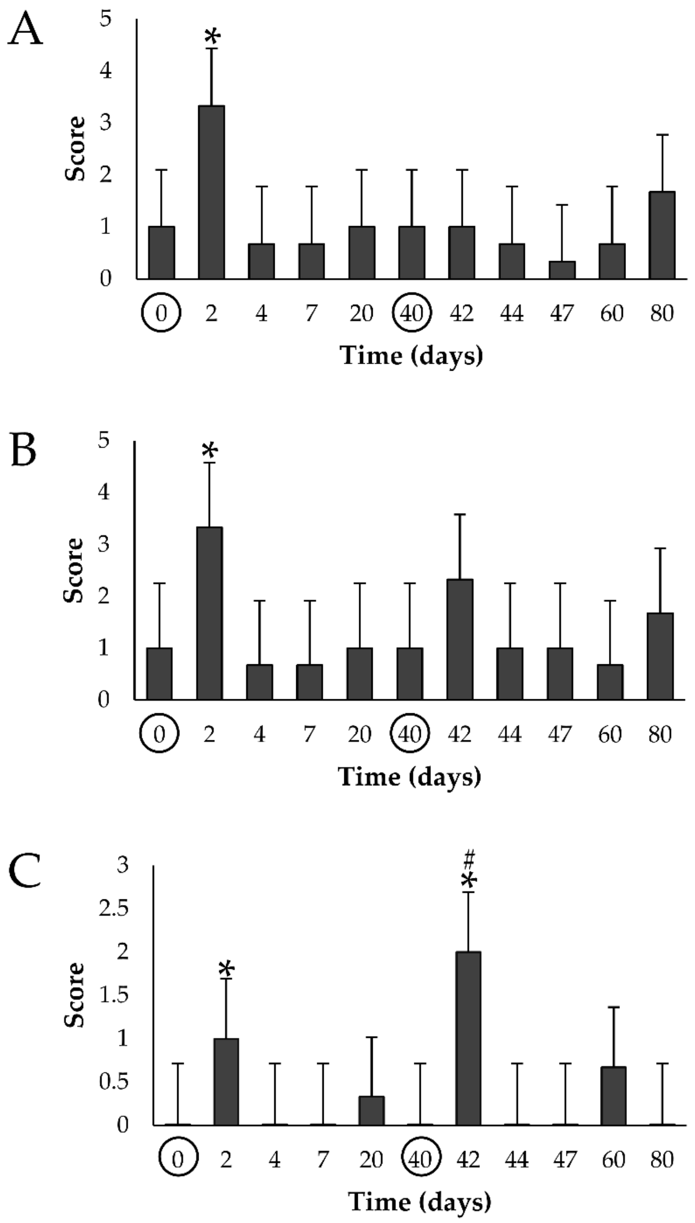

Figure S4. Scores for questions 11 (A), 12 (B) and 21 (C) as a function of time. The treatment (Lyosecretome or placebo) was administered on day 0 and day 40 (circled). Multifactor ANOVA, mean values ± LSD (n = 3). * $p<0.05$ vs t = 0; # $p<0.05$ vs t = 2.

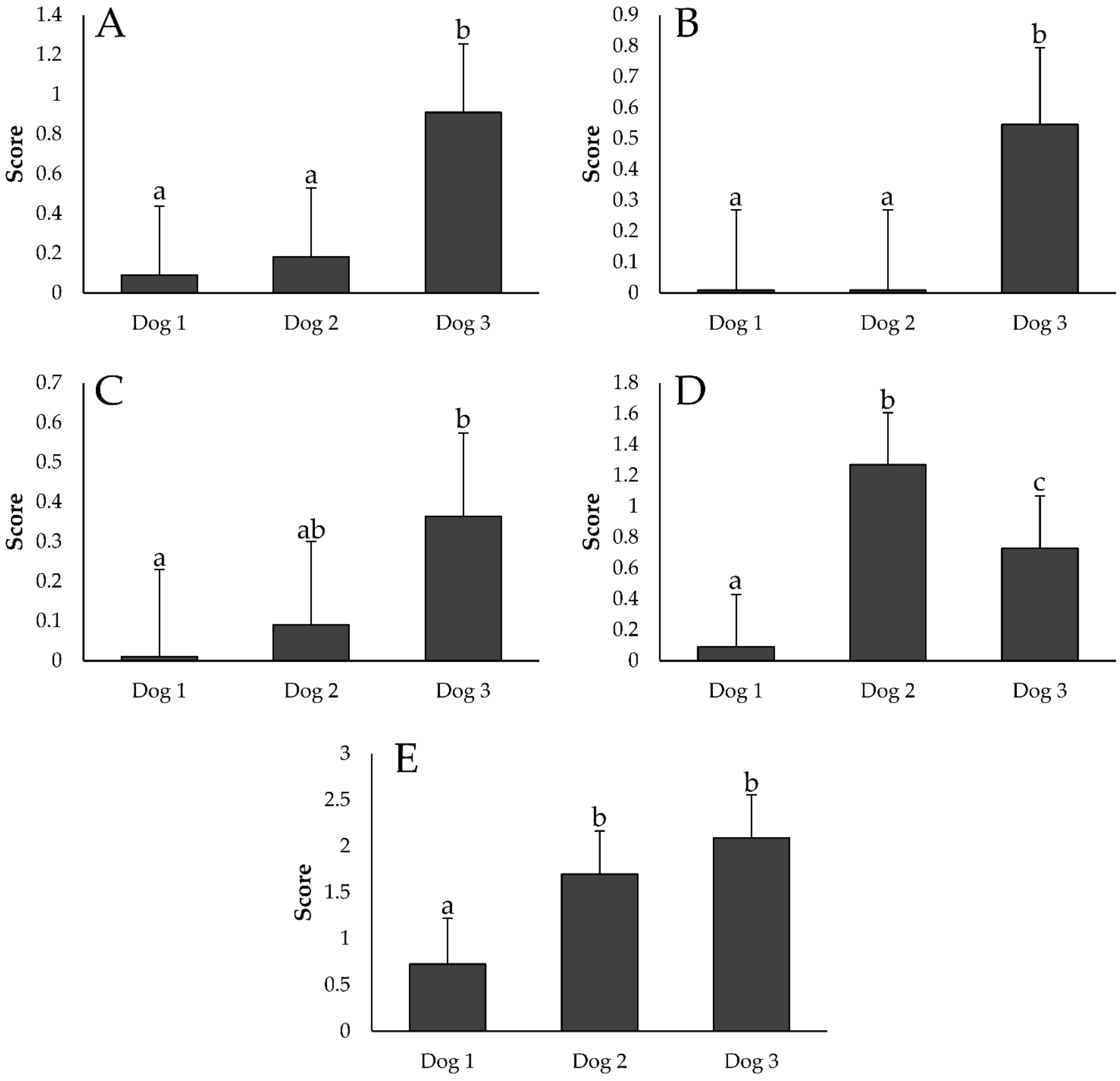

Figure S5. Scores for questions 4 (A), 5 (B), 9 (C), 14 (D) and 16 (E) for each participant. Multifactor ANOVA, mean values ± LSD (n = 3). Different letters (a-h) indicate a significant difference between the means ($p<0.05$), while the same letters indicate no significant differences ($p>0.05$).
